# Born Normalization for Fluorescence Optical Projection Tomography for Whole Heart Imaging

**DOI:** 10.3791/1389

**Published:** 2009-06-02

**Authors:** Claudio Vinegoni, Daniel Razansky, Jose-Luiz Figueiredo, Lyuba Fexon, Misha Pivovarov, Matthias Nahrendorf, Vasilis Ntziachristos, Ralph Weissleder

**Affiliations:** Center for Systems Biology, Harvard Medical School; Center for Systems Biology, MGH - Massachusetts General Hospital; Institute for Biological and Medical Imaging, Technical University of Munich and Helmholtz Center Munich

## Abstract

Optical projection tomography is a three-dimensional imaging technique that has been recently introduced as an imaging tool primarily in developmental biology and gene expression studies. The technique renders biological sample optically transparent by first dehydrating them and then placing in a mixture of benzyl alcohol and benzyl benzoate in a 2:1 ratio (BABB or Murray s Clear solution). The technique renders biological samples optically transparent by first dehydrating them in graded ethanol solutions then placing them in a mixture of benzyl alcohol and benzyl benzoate in a 2:1 ratio (BABB or Murray s Clear solution) to clear. After the clearing process the scattering contribution in the sample can be greatly reduced and made almost negligible while the absorption contribution cannot be eliminated completely. When trying to reconstruct the fluorescence distribution within the sample under investigation, this contribution affects the reconstructions and leads, inevitably, to image artifacts and quantification errors.. While absorption could be reduced further with a permanence of weeks or months in the clearing media, this will lead to progressive loss of fluorescence and to an unrealistically long sample processing time. This is true when reconstructing both exogenous contrast agents (molecular contrast agents) as well as endogenous contrast (e.g. reconstructions of genetically expressed fluorescent proteins).

**Figure:**
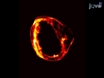


## Protocol

### Imaging Procedure

The experimental setup is shown in detail in Figure 1. A light source LS serves both as illumination for absorption measurements and as excitation source for fluorescence measurements and it is filtered with a narrow band pass interference filter BPF. A set of fixed and variable neutral density filters ND combined with the presence of an automatic shutter S allow to control the amount of light on the sample and to keep it low enough to prevent any photobleaching. Uniform sample illumination is achieved by using a beam expander BE with a combined two lenses Galilean telescope and a diffuser. The sample S is immersed in the clearing solution and is held in place on a holder and rotated along its vertical axis by way of a high speed rotation stage (Newport, PR50) with an absolute accuracy of 0.05 degrees. Three distinct manual controllers allow for the sample vertical axis to be tilted and adjusted in its orthogonal plane. The transillumination signal is directly detected by the CCD camera; the fluorescence signal is filtered through a narrow band-pass interference filter coupled with a longpass filter (Omega. Optical, Brattleboro, VT) and then collected with a telecentric lens TL. Telecentric lenses offer the advantage of providing unique features that make them ideal for optical projection tomography. In fact the presence of an aperture stop located within the lens assembly at the focal point of the lens assures that rays, that make the image of the aperture stop, will travel parallel to the optical axis virtually eliminating any perspective distortion and providing the same magnification for multiple planes within the telecentric depth.

### Sample Preparation

The following procedure is followed in order to fix the tissue/organs

Samples are fixed in PFA for 8 hours at 4C.
Sample is then washed in PBS for 15 minutes
The sample is embedded in a 0.8% Agarose block
Agarose block is dehydrated through a series of 20% to 100% of Ethanol solutions. This dehydrating ethanol series procedure helps in avoiding any asymmetric shrinkage of the sample.
Finally the sample is incubated 10 hours in 100% ethanol in order to remove any water content from the sample.
Put the sample in a solution 1:2 of Benzyl Alcohol and Benzyl Benzoate for the time necessary for the sample to clear. Note that the time can vary considerably from sample to sample from a few hours to several days.
Ethanol in minimal traces can be present at the end of the clearing process giving rise to sever artifacts in the reconstructions due to thermal motion of the alcohol within the clearing solution itself. In order to avoid this problem, a second clearing cycle in recommended.
Point 4 and 5 should be performed in the dark to avoid any bleaching of fluorescent contrast agents or proteins.

### Heart Sample Preparation

It is important to remove any blood content from any tissue prior to be imaged in order to avoid high absorption artifacts in the reconstructions. The following procedure can be followed.

Anesthetize the mouse with an intraperitoneally (IP) injection of a mix of ketamine (90 mg/kg) and xylazine (10mg/kg).Note: "From this point forward keep the sample protected from light."
Inject 50 U of heparin (IP) and five minutes later, perform a longitudinal laparotomy.
Open the left renal vein (LRV) and infuse 20ml of saline solution into the IVC.
The agarose block should be dehydrated with four, one hour incubations, in 20%. 40%,60%, and 80% ethanol, and then finally, samples are incubated in 100% ethanol for 10 hours (overnight).
Heart is then fixed following the general sample preparation procedureNote: "From this point forward keep the sample protected from light."

### Absorption Reconstructions

Reconstruction of optical absorption in the absence of scattering are in general analogous to X-CT and can be obtained using a common filtered Radon backprojection algorithm. The absorption images are then taken by a CCD camera in transillumination over 360 projections with a 1 degree angle along the vertical axis. It is convenient to align the vertical axis of the sample parallel to the column of pixels of the CCD.

Place the block of agarose into a chamber filled with clearing solution
Illuminate sample with a collimated beam for both excitation and transmission measurements
Rotate sample over 360 projections with a 1 degree angle.
Acquire images in transillumination at both absorption (intrinsic) and fluorescence wavelengths.
Place a shutter to avoid continuous illumination and reduce bleaching.
Use a filtered Radon backprojection algorithm to tomographically reconstruct the sample

### Fluorescence Reconstructions

The algorithm used for the absorption reconstructions is not valid for reconstruction of fluorescence distribution and, if not taken into account, the absorption contribution will lead to severe artifacts.

Proceed as indicated in the “Absoprtion Reconstructions” section.
Acquire simultaneously both absorption and fluorescence measurements
Combine the data into the normalized Born field images
Write down equations of the forward model of light propagation for intrinsic and fluorescence wavelengths.
Calculate weight matrix on a discretized mesh for Green’s functions of the forward model
Invert the weight matrix and multiply it by the detected Normalized Born field images to obtain reconstruction of fluorescence distribution in the object
